# Patterns of ecstasy use amongst live music event attendees and their opinions on pill testing: a cross sectional study

**DOI:** 10.1186/s13011-020-00295-1

**Published:** 2020-08-05

**Authors:** Madeleine Southey, Ashwini Kathirgamalingam, Benjamin Crawford, Rohan Kaul, Jack McNamara, Franklin John-Leader, Jenny Heslop, Sabrina Winona Pit

**Affiliations:** 1 University Centre for Rural Health, School of Medicine, Western Sydney University, 62 Uralba Street, PO Box 3074, Lismore, NSW 2480 Australia; 2 Faculty of Medicine and Health, University Centre for Rural Health, Lismore, Australia; 3 Harm Reduction and Health Promotion Programs, HIV and Related Programs (HARP), North Coast Public Health, Mid-North Coast Local Health District, Lismore, Australia

**Keywords:** Pill testing, Ecstasy, MDMA, Harm reduction, Festivals, Public health

## Abstract

**Background:**

Pill testing services could potentially be used to reduce drug-related harm. This study aims to identify patterns of ecstasy use among live music event attendees; explore the opinions and potential usage of illicit pill testing programs and examine factors associated with the likelihood of still taking a pill containing a potential harmful substance.

**Methods:**

A cross-sectional survey was completed by 760 people attending a major Australian live music event in 2017.

**Results:**

The most commonly used drug in the last 12 months was ecstasy (73.9%). About 5% of people who use drugs had sought medical attention due to consumption of ecstasy. People who use drugs agreed “a lot” that pill testing should be provided for free at live music events (82.2%) and that it should be combined with harm reduction advice (62.9%). Additionally, 32% of all participants agreed ‘a lot’ that they would be more likely to take illicit drugs at a music festival if pill-testing services were present. However, if people perceived that a harmful substance was detected in their drugs after using a pill testing service, 52.3% of people who have used illicit drugs reported that they would ‘not at all’ be likely to still consume the drug. They also reported that they would still take a pill if testing demonstrated the presence of unintended MDMA-type substances (70.3%), amphetamines (31.2%) or ketamine (27.8%). Multivariate analyses demonstrated that only increased frequency of ecstasy use was significantly associated with taking a pill despite pill testing services detecting a harmful substance. Gender, age, alcohol and previously seeking ecstasy-related medical attention were not associated in the multivariate analyses.

**Conclusions:**

A high proportion of live music attendees consume alcohol and ecstasy. Both people who have and who have not used illicit drugs support the implementation of pill testing services. People reported they would change their consumption patterns according to the results given by pill testing services. The findings may be used to stimulate public debate, and assist drug and alcohol policy makers in the implementation of harm minimisation strategies such as combining pill testing services with harm reduction advice.

## Background

Ecstasy use and its associated risk profile has become increasingly relevant in western society, with Australia ranking as the highest per capita consumer of the drug in the world as per the 2014 United Nations World Drug Report [1]. A survey conducted by the Ecstasy and Related Drugs Reporting System (EDRS) in 2017 revealed it to be the most popular illicit drug for 36% of respondents who had used psychostimulants in the last 6 months [2]. Trends in the place of consumption indicate that the majority of ecstasy use occurs at public entertainment events and venues, with live music events being the 2nd most frequent location for ecstasy consumption in Australia [2]. From a wider sample of the Australian population 14 years and over, 400,000 people (2.2%) had used ecstasy in the last 12 months, whilst general illicit drug use in the same time period was reported amongst 15.6% of respondents [3]. The use of such illicit drugs remains highest in the 20–29 age category (32%) [3], with the median age of ecstasy use being 28 years [3]. Illicit drug use costs the Australian economy $8 billion annually; this includes losses in productivity, health care costs and those associated with combatting trafficking and production [4].

Ecstasy is a prohibited substance as per The Drug Misuse and Trafficking Act 1985 [5]. Consequent to the drug primarily being sourced through illegal avenues, there exists a lack of regulation and quality assurance associated with its production, creating significant risk to consumers who are often exposed to adverse side effects from added substances and impurities. As of 2016, almost 9.3% of the participants surveyed who had taken drugs had been the victim of an ‘illicit drug related incident’, which ranged from a mild reaction to serious adverse health outcomes; injury or death [3].

Many studies internationally, such as Israel [6] and the UK [7, 8], have explored the morbidity and mortality associated with ecstasy, including headaches, dehydration, agitation, hypertension, hyperthermia, loss of consciousness, cerebral oedema, serotonin syndrome, multi-organ failure and in some instances, sudden death [6–8]. Overdoses involving ecstasy pills and the added substances they contain have also been reported amongst users, with 58% of stimulant related overdose respondents attributing their overdose to the consumption of ecstasy in 2016–2017 [2].

Drug markets are not stable and vary per country. A 2006 U.S. study found that, based on testing the content of ecstasy, a large proportion of ecstasy sourced from users (46%) did not contain any of its active ingredient; 3,4-Methylenedioxymethamphetamine (MDMA), and 15% of those tested contained a mixture of MDMA and other miscellaneous substances. The most common contaminant being Methylenedioxyamphetamine (MDA), methamphetamine, caffeine, dextromethorphan (DXM) and pseudo-ephedrine [9]. There were other substances detected including ketamine, dimethoxyamphetamine and heroin, albeit in a small percentages [9]. A 2012 Dutch study found that the occurrence of adverse events associated with ecstasy use, such as palpitations, agitation, hyperthermia and seizures could be attributed to the presence of contaminants such as MDA and meta-chlorophenylpiperazine (mCPP) [10]. Australia Drug Trends 2017 [2] also found increased availability of more potent forms of ecstasy when compared to previous years, raising concerns of increased potential harm for users.

These underlying dangers mean some organisations advocate for pill testing. For example, the Australian Drug Law Reform Foundation of Australia, have lobbied for legislative changes that allow pill testing services at Australian live music events as a means of harm minimisation [11]. The principle of harm minimisation formed the basis of the Australian National Drug Strategy since 1985. The harm minimisation framework encompasses three pillars: supply reduction, demand reduction and harm reduction, [12]. Pill testing falls under the principle of harm reduction, which Harm Reduction International defines as ‘*policies, programs and practices that aim to reduce the harms associated with the use of psychoactive drugs in people unable or unwilling to stop’* [13].

Internationally, many countries throughout Europe have existing pill testing facilities or ‘drug checking services’ which have been legislated for and instituted at festivals over the last 25 years as a harm minimisation strategy [14–16]. Success has been seen in various areas from implementation of this harm reduction strategy, including rapid on-site identification of contaminants and purity, identification of contaminated pill presses or batches [14], facilitation of contact between health services and ecstasy users [15], as well as serving as the basis for effective public warning systems in generating awareness of circulating harmful batches [15, 16].

Regularly, there is media attention surrounding pill testing at live music events which is presented by reporters and include the views of predominantly authority groups and political leaders on the topic [17, 18]. The at-risk population likely to utilise pill testing services appear to be underrepresented, and underreported. Few studies have examined pill testing in the context of Australian ecstasy and illicit drug usage, although the evidence is increasing. Previous papers have analysed the accuracy of pill testing, as seen in an Australian study conducted in 2005 by Camilleri & Caldicott [19]. More recently, a few Australian studies have assessed public opinion on pill testing. An online survey conducted in 2013 by the National Drug and Alcohol Research Centre [20] revealed 82.5% of respondents supported the implementation of a pill testing service. However, a much higher proportion of people in this survey had used ecstasy, with 47.7% asserting they had consumed ecstasy [20] versus the 8% found in National surveys across Australia [3]. Although the results represented the opinions of the Australian youth on this issue, the study only ascertained whether the individuals were in support of or against pill testing. There is a need to explore the role and expectations of pill testing services and their value in harm reduction at Australian live music events. A 2016 study investigated the perceptions of music festival attendees in detail and showed similar support for pill testing services with 86.5% of participants believing that pill testing services could help reduce harm [21]. Similar results were found by another recent study conducted by Barratt et al., whereby it was found that 94% would utilise a pill testing service at a festival [22]. This study builds further on the 2016 work conducted by Day et al. [21]. Since the 2016 survey [21], there have been a number of high profile deaths in Australia at live music events from drug overdose [23], which may have potentially influenced the opinions of live music festival attendees. Additionally, this study will focus on the opinions of ecstasy users in particular.

This paper aims to:

1. Identify characteristics and patterns of Ecstasy/MDMA use among live music event attendees
2. Explore the opinions of live music event attendees on pill testing programs
3. Identify the proportion of people that have used illicit drugs that would still take a pill after results of pill testing show the presence of unintended drugs or substances
4. Examine among people who have used illicit drugs which factors are associated with the likelihood of still taking a pill despite a pill testing service detecting a harmful substance in the pill.

## Methods

### Study design

A cross-sectional survey was conducted.

### Setting, participants and recruitment

Eligible participants were those aged over 18 attending a three-day large mixed-genre live music event in New South Wales in 2017. Surveys were distributed by five members of the research team throughout the course of the festival between 9 am and 12 pm each day. The five members were in their early twenties and consisted of two females and three males. The rationale for surveying between these times was to avoid intoxicated attendees later in the day. Potential participants were approached face-to-face and invited to complete the paper-based survey. Researchers were equipped with RSA (Responsible Service of Alcohol) skills to recognise those who may have been under the influence of alcohol and these individuals were excluded from the survey. Participants were given a Participant Information Statement and were told what was involved and what the study aims were. The survey was completed anonymously to ensure confidentiality. No identifying information was collected to ensure anonymity of participants. A self-completed survey format was used rather than an interview format, to minimise any discomfort regarding the sensitive nature of the questions. Completion of the survey was taken as consent.

### Outcome measures

The survey was adapted from the 2016 survey by Day et al. [21] The study by Day et al. was conducted at the same festival the year prior to the current study and included the same target population. Additionally, similar recruitment methods were used for both surveys. The 2016 survey was completed by 625 people. The 2017 survey included additional questions based on current literature and expert opinion (See Additional file 1). All participants completed a core set of questions regarding demographics, employment and study, sexual orientation, relationship status, alcohol consumption and Likert scale questions where participants were able to choose to what extent they agreed with certain statements about pill testing and its potential effectiveness at live music events. Alcohol consumption was assessed using questions from the World Health Organisation Alcohol Use Disorders Identification Test: The AUDIT-C [24]. Attitude questions towards pill testing were derived from a previous study [21]. The attitude questions were preceded with the following statement: *“Pill testing services could test drugs by taking a small sample. A reagent testing kit is used to test this sample, gathering information on its contents. The user can then be informed of the presence of potentially harmful ingredients/substances.”* An open-ended question was added asking whether patrons had any concerns about pill testing at music festivals.

The second section was only completed by people who reported ever having taken illicit drugs. Questions regarding the use of illicit drugs were derived from the National Drug Strategy Household Survey [3]. Questions measured type of drug used in the last 12 months, detailed questions about ecstasy use, how concerned they were about their health in relation to content and/ or purity of illicit drugs and if they would utilise pill testing services. To identify how participants perceived that pill testing services would influence their behaviour, participants were asked a general question: “*If a harmful substance was detected in your drugs using the pill testing service, how likely would you be to still consume them*?” with answers ranging between ‘not at all’, ‘a little’, ‘somewhat’ and ‘a lot’. Participants were also asked a more specific question: “*Would you still take a drug if the results of a drug testing kit/ pill testing service indicated the presence of the following unintended drugs?”.* The response options were: ‘*yes*’, ‘*no*’ or ‘*don’t know’*. (See Additional file 1 for full list of drugs*).* Open-ended questions asked about other substances individuals had consumed at the same time as ecstasy and the amount they would be willing to pay for the service if it were not free. Two pilot testing sessions were conducted during survey development to test the data collection tool on groups of young adults (*n* = 15 and *n* = 10). The 1st pilot-tests was conducted among students and young people directly known to the researchers. This group was chosen because the music festival is attended mainly by young people, and thus represented the target population. The second pilot test was conducted among university students.

### Statistical methods

Chi-square tests were used to determine whether alcohol use was associated with the likelihood of altering drug taking behaviours based on pill testing results as well as for determining the association between those that had used illicit drugs or not with attitudes towards pill testing. *P*-values less than 0.05 were considered to indicate statistical significance.

Among MDMA users, the question *“If a harmful substance was detected in your drugs using the pill testing service, how likely would you be to still consume them?”* was categorised into: “not at all” versus “would consider”. The “would consider” group included those that had answered “a little”, “somewhat” or “a lot”. Logistic regression was used to calculate crude and adjusted odds ratios to identify which factors were significantly associated with MDMA users consuming a pill even if a harmful substance was detected in their drugs after using a pill testing service. SPSS Version 22 was used.

## Results

### Socio-demographics and drug use characteristics and patterns

The survey was completed by 760 people between the ages of 18 and 56 and took approximately 5 min to complete.

#### Sample description

The majority of participants were male (55%), aged 18–21 (59.6%), single (50.8%), heterosexual (91.3%), employed (92.6%) and/or fulltime students (40.6%) (Table 1). Of all participants, 40.9% reported using alcohol 2–3 times per week. Most participants reported having used illicit drugs (83.9%) in their lifetime and the most commonly used drugs in the last 12 months were ecstasy/MDMA (73.9%), cannabis (64.3%) and cocaine (45.5%) (Table 2). Some people who have used illicit drugs reported having time off work due to illicit drug use (16.7%) and just over one in four reported that they had gone to work or study despite feeling that they should have taken sick leave due to their use of illicit drugs (Table 2).

**Table 1:** Participant characteristics (*N* = 760)

	2017%	Comparison data 2016^**a**^%
**Gender (*n*** **= 758)**		
Male	55.0	39.0
Female	43.7	61.0
Other	1.3	0.5
**Age (*n*** **= 754)**		
18–19	28.2	–
20–21	31.6	–
22–23	22.3	–
24+	17.9	–
**Relationship status (*n*** **= 756)**		
Married/ de facto	4.8	1.6
Single	50.8	56.8
In a relationship	43.2	41.0
Separated/divorced/widowed	0.8	0.6
**Sexuality (*n*** **= 750)**		
Heterosexual	91.3	90.4
Homosexual	2.7	2.6
Bisexual	4.8	5.9
Other	1.2	1.1
**Employment status (*n*** **= 757)**^**b**^		
Full time student	40.6	48.9
Part time student	5.8	7.2
Employed	92.6	87.5
Unemployed	1.6	12.5
Other	0.5	0.5
**Hours worked per week (*n*** **= 744)**		
0–10	13.4	–
11–20	21.1	–
21–30	17.2	–
31–40	35.4	–
41+	12.9	–

^a^Source: Day et al., 2018 [21]; ^b^ Multiple responses possible

**Table 2:** Prevalence drug and alcohol use among participants (N = 760)

Variable	2017%	Comparison data 2016^**a**^%
***ILLICIT DRUGS***		
**Ever used illicit drugs (n = 758)**	83.9	–
**Drugs used in the last 12 months (*n*** **= 747)**		
Ecstasy/MDMA	73.9	59.8
Cannabis	64.3	63.9
Cocaine	45.5	34.1
Hallucinogens	25.3	20.2
Amphetamine	21.4	18.4
Recreational Pharmaceutical drugs	17.4	13.6
Inhalants	17.4	8.4
Ketamine	12.9	12.5
Methamphetamine	10.0	4.7
Synthetic cannabis	9.0	4.8
GHB	2.9	2.3
Steroids	1.9	1.7
Other	0.9	–
**Taken time of work/ study in the last 12 months due to illicit drug use (*n*** **= 617)**	16.7	
**Gone to work/ study despite feeling they should have taken sick leave due to illicit drugs use (*n*** **= 610)**	27.5	
***ALCOHOL***		
**Frequency of alcoholic drink (n = 758)**		
Never	0.1	–
Monthly or less	8.2	–
2–3 times per month	38.5	–
2–3 times per week	40.9	–
More than 4 times per week	12.3	–
**Standard drinks per drinking session (*n*** **= 753)**		
1 to 2	10.0	–
3 to 4	12.4	–
5 to 6	22.2	–
7 to 9	23.6	–
More than 10	31.9	–
**Frequency > 6 standard drinks on one occasion (n = 754)**		
Never	0.4	–
Less than monthly	13.5	–
Monthly	39.1	–
Weekly	45.1	–
Daily or almost daily	1.9	–

^a^Source: Day et al., 2018 [21]

#### Comparison with 2016 sample

The 2017 sample differed when compared to the Day et al. 2016 study sample [21]. In 2016, 39% were men [21], however in the 2017 sample there was a higher proportion of males to females. Relationship status was similar in both groups as was the spread of sexuality. Unemployment rates were higher in 2016 (12.5%) [21] compared to 2017 (1.6%). Table 2 compares drug use in 2017 compared to what was found in 2016. Reported drug use in the 2017 sample was higher for every substance than in 2016. This was especially evident for ecstasy/MDMA with 73.9% in 2017 compared with 59.8% in 2016 [21], as well as cocaine use, 45.5% in 2017 compared to 34.1% in 2016 [21]. Use of methamphetamines and inhalants in 2017 was just over double what it was in 2016 [21].

Among those who reported having used illicit drugs in the past (n = 636), a high proportion (87.8%) reported having consumed MDMA/ecstasy at a live music event (Table 3). Of this sample of MDMA/ecstasy users at festivals (*n* = 548) the majority (82%) reported mixing MDMA/ecstasy with other substances. The most common substances that were mixed with MDMA/ecstasy included alcohol (46.8%), cannabis (31.2%) and cocaine (24.5%). The majority of MDMA/ecstasy users reported using monthly (32.1%) or every 6 months (30.9%) and 4.6% reported having to seek medical attention.

**Table 3:** Characteristics of MDMA/ Ecstasy use in 2017 at live music events among participants who have ever used illicit drugs (*N* = 636)

	Percent
**Ever used MDMA/ ecstasy at a life music event (*n*** **= 636)**	
Ever consumed MDMA/ecstasy at a live music event	87.8
Ever mixed MDMA with other substances at a life music event	82.0
**Most common substances mixed among MDMA users (*n*** **= 548)**	
Alcohol	46.8
Cannabis	31.2
Cocaine	24.5
Hallucinogens	17.4
Tobacco	9.6
Amphetamine	9.6
Ketamine	6.5
Inhalants	4.4
Valium	1.5
Amyl Nitrate	0.8
Viagra	0.4
GHB	0.4
**Amount taken (mg) at one time at a music festival (n = 548)**	
1–100	20.8
101–200	31.7
201–300	19.1
301–400	8.4
401–500	6.0
501+	14.1
**Frequency of MDMA/ecstasy use (n = 548)**	
Fortnightly or more	12.9
Monthly	32.1
6 monthly	30.9
Yearly or less	16.0
Never	8.1
**Medical attention sought due to MDMA/ecstasy (n = 548)**	4.6
**Price users are willing to pay for pill testing services? (*n*** **= 497)**	
$0	14.6
$1–$5	33.6
$6–$10	31.3
$11–$15	5.8
$16+	14.7

### Opinions of live music event attendees on pill testing programs

Table 4 demonstrates all participants’ attitudes towards pill testing services. The majority of participants agreed ‘a lot’ (79.2%) or ‘somewhat’ (15.5%) that pill testing services should be provided for free at live music events. Conversely, the majority disagreed with providing pill testing services at a cost responding with ‘not at all’ (38.9%) or ‘a little’ (22.8%). Most agreed ‘a lot’ (75.2%) and ‘somewhat’ (19.1%) that pill testing services could help people that use drugs to seek help and reduce harm and similarly ‘a lot’ (62.5%) and ‘somewhat’ (25.9%) agreed that pill testing services should be combined with harm reduction advice.

**Table 4:** Attitudes towards pill testing in 2017 (*N* = 760)

	Total %	People who use illicit drug %	People who do not use illicit drug %	Chi-square, df^**b**^, p- Value	Comparison data 2016^**a**^	Percent
**Pill testing should be provided for free at live music events (*n*** **= 756)**^**c,d**^						
Not at all	2.9	3.0	2.5	χ = 25.72,df = 3, *p* < 0.001	Not at all/ a little	11.8
A little	2.4	1.7	5.7			
Somewhat	15.5	13.1	27.9		Somewhat/ a lot	86.2
A lot	79.2	82.2	63.9			
**Pill testing should be provided at a cost at live music events (n = 754)**^**c**,**d**,e^						
Not at all	38.9	37.8	44.6	χ = 4.0, df = 3, *p* = 0.26	Not at all/ a little	32.5
A little	22.8	22.6	24.0			
Somewhat	25.1	26.4	18.2		Somewhat/ a lot	67.5
A lot	13.3	13.3	13.2			
**Pill testing services could help drug users seek help to reduce harm**^**d**^**(*n*** **= 743)**						
Not at all	2.4	2.2	3.3	χ = 18.20,df = 3, p < 0.001	Not at all/ a little	13.5
A little	3.2	2.7	5.8			
Somewhat	19.1	16.9	30.8		Somewhat/ a lot	86.5
A lot	75.2	78.2	60.0			
**Pill testing services should be combined with harm reduction advice (n = 753)**^**d**^						
Not at all	3.2	3.2	3.3	χ = 0.22,df = 3, *p* = 0.975	Not at all/ a little	15.1
A little	8.4	8.4	8.3			
Somewhat	25.9	25.6	27.5		Somewhat/ a lot	84.9
A lot	62.5	62.9	60.8			
**Drug sellers may use the service as a quality control mechanism (*n*** **= 751)**						
Not at all	10.5	10.2	12.4	χ = 4.73,df = 3, *p* < 0.192	Not at all/ a little	31.4
A little	16.6	15.9	20.7			
Somewhat	30.4	29.8	33.1		Somewhat/ a lot	68.6
A lot	42.5	44.1	33.9			
**I would be more likely to take illicit drugs at a music festival if pill testing services were present (n = 750)**						
Not at all	25.3	23.5	35.3	χ = 15.46,df = 3, *p* = 0.001	**–**	
A little	18.0	17.4	21.0			
Somewhat	24.7	24.4	26.1			
A lot	32.0	34.7	17.6			

^a^ Source: Day et al, 2018 [21]; ^b^*df* degrees of freedom; ^c^In 2016, the word ‘festivals’ was used instead of ‘live music event’; ^d^ In 2016, the words ‘drug checking services’ was used instead of ‘pill testing services’; ^e^ In 2016, the question was worded as follows: “If not free, drug checking services should be provided AT-COST at festivals”

Compared to people who do not use illicit drugs, people who use drugs were found to be significantly more likely to support pill testing being provided for free at live music events, more likely to believe that pill testing services could help people who use drugs to seek help and reduce harm, and were significantly more likely to take illicit drugs at live music events if pill testing was provided.

#### Comparison with 2016 sample

There was a small difference in the responses to whether pill testing should be provided for free at live music events with a higher response for those agreeing “somewhat” or “a lot” in our survey (94.7%) compared to the 2016 survey (86.2%) [21]. There was a larger difference in the responses to whether pill testing should be provided at a cost at live music events with a lower response for those agreeing “somewhat” or “a lot” in our survey (38.4%) compared to the 2016 survey (67.5%) [21]. Compared to 2016, participants in 2017 reported slightly higher levels of agreeing ‘somewhat’ or ‘a lot’ with the statement ‘Pill testing services could help drug users seek help to reduce harm’ (94.3% in 2017 versus 86.5% in 2016). Similar proportions in 2017 (88.4%) and 2016 (84.9%) of participants agreed ‘somewhat’ or ‘a lot’ that ‘Pill testing services should be combined with harm reduction advice’. Finally, similar proportions in 2017 (72.9%) and 2016 (68.6%) agreed ‘somewhat’ or ‘a lot’ that drug sellers may use the service as a quality control mechanism.

Participants who had previously used illicit drugs (n = 636) were asked about their opinions on their pills (Table 5). Most participants agreed ‘somewhat’ (38.3%) or ‘a lot’ (20.9%) that they were concerned about the content and/or purity of their illicit drugs in terms of their health. When asked if they were likely to consume their drugs if a pill testing service found they contained a harmful substance, a large proportion of respondents were unlikely to, with responses of ‘not at all’ (52.3%) and ‘a little’ (25.9%) being the most prevalent. Responses were mixed in regards to how likely participants currently are to test the content and/or purity of their illicit drugs. A high proportion of participants agreed ‘somewhat’ (17.1%) or ‘a lot’ (73.3%) that they were likely to use a free pill testing service if it was provided at live music events.

**Table 5:** Behaviour among people that ever used illicit drugs (*N* = 636)

	% (n)	Comparison data 2016^**a**^	Percent
**In terms of your health how concerned are you about the content and/or purity of the illicit drugs you take (*n*** **= 618)**			
Not at all	13.8	Not at all/ a little	47.2
A little	27.0		
Somewhat	38.3	Somewhat/ a lot	52.8
A lot	20.9		
**If a harmful substance was detected in your drugs using the pill testing service, how likely would you be to still consume them? (n = 618)**			
Not at all	52.3	–	
A little	25.9		
Somewhat	16.3		
A lot	5.5		
**Currently, how likely are you to attempt to find out about content and/or purity of the illicit drugs you intend to take? (*n*** **= 615)**			
Not at all	19.8	–	
A little	26.7		
Somewhat	27.5		
A lot	26.0		
**How likely is it that you would use a free pill testing service? (n = 615)**			
Not at all	3.1	–	
A little	6.5		
Somewhat	17.1		
A lot	73.3		

^a^ Source: Day et al, 2018 [21]

#### Comparison with 2016 sample

When compared to 2016 survey data, there was a slight increase in individuals who reported being ‘somewhat or ‘a lot’ concerned in terms of their health about the content and/or purity of illicit drugs taken from 52.8% in 2016 [21] to 59.2% in 2017. In 2016, 13% responded that they would not at all be likely to utilise a free pill testing service [21] compared to only 3.1% of individuals in 2017. The majority of individuals in 2016 and 2017 responded that their likelihood of using a pill testing service was ‘a lot’ however this was higher in the 2017 cohort (73.3%) compared to 2016 data where 54.4% responded it was ‘highly likely’ they would use a free drug checking service [21].

### Considerations of still taking a drug if the results of a drug testing kit/ pill testing service indicated the presence of unintended drugs

Table 6 shows whether pill testing would influence drug consumption among people who use drugs, if it showed that the pill had various unintended drugs. A majority of participants indicated they would still take the drug if it contained MDMA-type substances (70.3%), this was followed by amphetamine (31.2%), ketamine (27.8%), opiates (17.8%), and methamphetamine (17%). The 2017 findings were very similar to the 2016 data, however there was an increase from 20.9% in 2016 [21] to 27.8% in 2017 of those who would still take a pill if it unintentionally contained ketamine.

**Table 6:** Proportion of people that have used illicit drugs that would still take a drug if the results of a drug testing kit/ pill testing service indicated the presence of unintended drugs (*n* = 636)

	Would take %	Would not take %	Don’t know %	Comparison data 2016^1^ Would take %
MDMA- type substances (MDA, MDE) (*n* = 622)	70.3	10.5	19.3	69.7
Amphetamine (n = 622)	31.2	36.8	32.0	32.9
Ketamine (*n* = 622)	27.8	42.1	30.1	20.9
Opiates (n = 622)	17.8	41.3	40.8	15.7
Methamphetamine (*n* = 622)	17.0	52.3	30.7	14.9
2C-B/C/I (*n* = 622)	6.4	43.1	50.5	7.9
DXM (*n* = 621)	6.0	44.4	49.6	7.0
No reaction (benign or unknown substances) (n = 622)	5.9	41.8	52.3	6.3
PMA/PMMA (n = 622)	3.9	44.2	51.9	3.8
Methylone (n = 622)	3.2	45.2	51.6	3.1
Butylone (*n* = 632)	3.1	45.2	51.8	1.8
DOB (n = 622)	2.9	44.1	53.1	3.1
Naphyrone (n = 622)	2.9	45.2	51.9	1.9
DOI (n = 622)	2.4	43.9	53.7	3.1
Other (n = 622)	2.1	42.3	55.6	2.5

^1^ Source: Day et al, 2018 [21]

### Factors associated with the likelihood of still taking a pill despite a pill testing service detecting a harmful substance in the pill

Table 7 demonstrates that the more frequently people drink, the more likely they are to consider taking a pill even if a harmful substance was detected in their drugs after using a pill testing service. Similarly, the more frequently people would consume more than six standard drinks in one session, the more likely they are to consider taking a pill even if a harmful substance was detected in their drugs after using a pill testing service.

**Table 7:** Alcohol and its association with the likelihood of still taking a pill despite a pill testing service detecting a harmful substance in the pill among people who use drugs (*N* = 636)

	Would not consider	Would consider	Chi-square, df^a^, p-value^b^
How often do you have a drink containing alcohol? (*n* = 618)			
Never	0.0	100.0	χ = 13.30, df = 4, *p* = 0.006
Monthly or less	62.8	37.2	
2–3 times per month	58.5	41.5	
2–3 times per week	49.4	50.6	
4+ times weekly	38.8	61.3	
Frequency of 6+ standard drinks in 1 session (*n* = 613)			
Never	0.0	100.0	χ = 12.03, df = 4, *p* = 0.010
Monthly or less	67.9	32.1	
2–3 times per month	53.3	46.7	
2–3 times per week	48.3	51.7	
4+ times weekly	33.3	66.7	

^a^*df* degrees of freedom ^b^ Exact values used due to small numbers

Table 8 demonstrates that those who drink alcohol more than four times a week are more likely to consume a pill even if a harmful substance was detected in their drugs after using a pill testing service (crude OR 1.935, 95%CI 1.116–3.357). Similarly, those using MDMA daily, weekly or fortnightly were more likely to consume a pill even if a harmful substance was detected in their drugs after using a pill testing service when compared to six monthly, yearly or one time users (crude OR 2.124, 95%CI 1.457–3.097). However, multivariate analyses demonstrated that only increased frequency of MDMA use was significantly associated with taking a drug despite pill testing services detecting a harmful substance after adjusting for all other variables.

**Table 8:** Crude and adjusted odds ratios and multivariate logistic regression among MDMA users that would consider taking a pill, even if a harmful substance was detected compared to those who would not take the pill

	%	***P*** value	Crude OR	95% CI		P -value	Multi-variate OR	95% CI	
				Lower	Upper			Upper	Lower
**Gender**									
Male	59.1	0.146	1.292	0.915	1.825	.158	1.298	.904	1.864
Female	40.9	–	1.000	–	–	–	1.000	–	–
**Age** per year (continuous)	–	0.245	0.974	0.931	1.018	.153	.965	.919	1.013
**How often do you have a drink containing alcohol?**									
Never/ monthly or less/ 2–3 times a month	42.3	–	1.000	–	–	.072	.578	.318	1.051
2–3 times a week	45.1	0.064	1.406	0.980	2.018	.206	.687	.383	1.230
4 or more times a week	12.6	**0.019**	1.935	1.116	3.357		1.000	–	–
**How often do you use MDMA?**									
Six month/ yearly/ once	49.6	**–**	1.000	–	–	**–**	1.000	–	–
Monthly	35.7	**0.002**	2.280	1.364	3.809	**.042**	1.775	1.022	3.082
Daily/weekly/fortnightly	14.6	**< 0.0005**	2.124	1.457	3.097	**.004**	1.785	1.203	2.647
**Have you ever had to seek medical attention due to taking MDMA?**									
Yes	4.8	**0.049**	2.349	1.003	5.498	.105	2.173	.850	5.555
No	95.2	–	1.000	–	–	–	1.000	–	–

## Discussion

Multivariate analyses demonstrated that only frequency of ecstasy use was significantly associated with taking a pill despite pill testing services detecting a harmful substance. Gender, age, alcohol and previously seeking ecstasy-related medical attention were not associated in the multivariate analyses with taking a pill containing harmful substance.

In univariate analyses, a correlation was found with the frequency of high risk or “binge” drinking and its association with the likelihood of still taking a pill despite a pill testing service detecting a harmful substance.

There was a steady increase in the proportion of people who would consider taking a pill, even if a harmful substance was detected in their drugs after using a pill testing service, in relation to their increasing frequency of hazardous drinking. Hazardous alcohol consumption, as well as the willingness to consume a pill that may harm the individual, reflect risk-taking behaviours. It is unsurprising therefore that individuals who partake in risk-taking, hazardous alcohol consumption may also be inclined to demonstrate risk-taking behaviour associated with their pill taking habits. The link between ecstasy consumption and risk-taking behaviour is well supported in the literature [25].

Analysis of trends in amount of ecstasy consumed by people that use illicit drugs revealed that whilst the proportion of people that had ever consumed ecstasy at a live music event was high (87.8%), 20.8% took a relatively low dosage of MDMA/ecstasy at any one time (100 mg or less). However, 65.2% of ecstasy users consumed between 101 and 500 mg of MDMA/ecstasy in one session and 14.1% consumed greater than 500 mg of MDMA/ecstasy in the same time period. These results reflect a group of people putting themselves at risk of adverse effects, given that the results of a study conducted in 2012 showed that detrimental reactions were more likely to occur at MDMA dosages greater than 120 mg [10]. It is worthy to note that the accuracy in reporting dosages ingested may be compromised by the fact that the actual MDMA content may differ from what is expected [9]. In keeping with the aforementioned high risk-taking behaviour seen among young festival goers, the frequency of ecstasy use was also relatively high; with 12.9% reporting use every 2 weeks or more and 32.1% reporting monthly use.

Our research reinforced many of the findings published by Day et al. [21]. Overall, all drug use appears to be higher in our sample than the 2016 sample. This may have been due to chance. The main demographic difference between the two samples was the gender ratio. There was also an increased reported unemployment level in the 2016 sample. All other demographics were comparable.

Another notable difference between the 2016 and 2017 studies was the increase in reported ecstasy use. Day et al. found that 59.8% of respondents had used ecstasy in the preceding 12 months [21], whereas in our study, 73.9% reported using ecstasy in that time period. This variation could potentially be explained by the difference in gender distribution between the two survey samples, with a 55% male majority in our study compared to a 61% female majority in the 2016 study [21], thus resulting in a difference in reported ecstasy use. Other confounding factors could be, for example, the frequency of attending live music events by participants but this was not measured in either study. The opinions of pill testing services were generally similar between 2016 and 2017. The most notable difference was that a lower proportion of 2017 participants agreed ‘somewhat’ or ‘a lot’ that pill testing should be provided at a cost at live music events (38.4% in 2017 versus 67.5% in 2016). This may be explained by the lower number of ecstasy users in the 2016 sample [21].

The findings of our study were further supported by Barrat et al. 2017 [22] who examined the acceptability of design features of pill testing services. They found that 94% of participants in their survey would use a pill testing service at music festivals or clubs. Whilst the question posed by Barrat et al. differed in that they queried whether participants would personally use the service versus our question regarding whether participants believed pill testing should be made available in a live music event setting, both showed a large majority in favour of the implementation of pill testing [22].

Compared to the 2016 survey, our data also showed a trend that indicates individuals are more concerned about their wellbeing when consuming illicit substances and increasingly likely to utilise pill testing services. In our survey only 3.1% of individuals responded that they would be ‘not at all’ likely utilise a free pill testing service compared with 13% in 2016 [21]. Almost three quarters of the individuals (73.3%) surveyed responded that their likelihood of using a pill testing service was ‘a lot’; when compared to 2016 data, where 54.4% of individuals responded it was ‘highly likely’ they would use a free pill testing service [21]. However, it should be acknowledged that this could be due to chance and we used convenience sampling. Future research, could examine changes in attitudes towards drug-checking over time and control for baseline differences.

Another important finding of our study is the extent to which individuals would heed the results of pill testing services if they were to be made available at Australian live music events. Over half of respondents (52.3%) answered that if a harmful substance was detected they would be ‘not at all’ likely to consume the drug. An additional 25.9% responded they would be ‘a little’ bit likely to consume the pill. This finding is poignant for policy makers as it indicates that individuals would not only utilise pill testing services, but would also trust and act upon the outcome of such testing, therefore altering consumption habits. However, still about half of the participants said they would consider taking the drug. When these findings are combined with increased individual investigation into the content and/or purity of their pills and the increased likelihood of utilising pill testing services should they be available, it is evident that people who use drugs are interested in participating in harm minimisation programs. These would have the potential to influence individual consumption patterns and potentially prevent individuals suffering adverse health outcomes. However, we also acknowledge that a variety of preventive actions are required for specific circumstances and different population needs. We also acknowledge that the risk of not detecting harmful substances with pill-testing services, and their reliability and accuracy remains.

Pill-testing has often been at the forefront of political debate in the last decade, particularly in terms of the intersections between drug policy, law enforcement praxis and perceptions of community safety. This context needs to be recognised. Our paper may be able to add to the debate about pill-testing, contributing valuable empirical data to inform knowledge and policy reform, whilst keeping the study limitations in mind. A concern of many opponents of pill testing is whether the availability of such a service would increase the prevalence of drug taking at live music events. We asked our participants how much they agreed with the statement “*I would be more likely to take illicit drugs at a music festival if pill testing services were present*”. We found that about one in four (23.5%) people who use illicit drugs and about one in three people who do not use drugs (35.3%) said they would be ‘not at all’ more likely to consume drugs at a festival if pill testing is available. Similarly, 34.7% of people who use drug versus 17.6% of those who do not use drug replied that they would be ‘a lot’ more likely to. This indicates that a proportion of attendees are more likely to consume drugs, if pill testing services were made available to them, but this proportion is smaller among those who do not take drugs. A 2011 Swiss study found that following the provision of drug checking facilities in Zurich, there was no increase in the frequency of consumption of most party drugs or in polydrug use (https://harmreductionjournal.biomedcentral.com/articles/10.1186/1477-7517-8-16).

### Limitations

Self-report and the inability to draw causal relationships were limitations. Due to the nature of the survey, people participating in illegal activities, such as the consumption of illicit substances, may have been reluctant to participate due to fears of police or festival security involvement, and prosecution for divulging this information. This means those partaking in illicit activities may be underrepresented in the data results of this study and represent non-responder bias. To combat this, the survey was specifically made anonymous so it was not possible to link the survey back to people who had completed the survey.

There may also have been a response bias, as it was a voluntary survey, and those who were in favour of the implementation of pill testing may have been more eager to participate.

Readers should bear in mind that sections of the survey relied heavily on individual’s knowledge of various illicit substances. Specific substances that were listed such as methylone or PMA/PMMA could also be considered a MDMA-type drug. Lack of knowledge of these substances, and their potential harm or limited awareness regarding what substances individuals were consuming may affect the accuracy of responses in these sections of the survey. This could skew the data for the drugs that individuals had consumed in the previous 12 months as well as the questions relating to the specific substances identified in pill testing, and whether the presence of these would affect their decision to take the drug. Participants’ perception of when a drug is considered harmful would also vary by participant. The survey did not provide a definition of harmful. Another related limitation was that any substances other than MDMA were a priori labelled as potentially harmful and thus could have been considered dangerous by the participant with limited knowledge.

Another factor that may have altered people’s opinions on pill testing was their knowledge of the purpose and processes involved in the testing. Those with limited knowledge on pill testing would have to rely on the explanation on the survey, and in the short amount of time that they would undertake the survey they would have to form many opinions about the process. This could be of concern as those with previous exposure to the idea of pill testing services would have had more time to reflect, question and form their opinions.

In an environment where individuals were consuming alcohol and illicit substances, severely intoxicated individuals partaking in the study could have altered the outcomes. Although this was minimised by recruiting participants in the morning and screening individuals for physical signs of intoxication (supported by RSA: Responsible Service of Alcohol training under the NSW Liquor Act 2007), it is a limitation that should be considered when interpreting the findings.

It was noted that the open-ended questions in the survey were poorly answered, resulting in considerable amounts of missing data. We therefore were not able to draw any conclusions from the open-ended questions.

## Conclusion

A high proportion of live music attendees consume alcohol and drugs, especially ecstasy. This suggests that they are a high-risk group that could potentially benefit from harm minimisation strategies. Both people who have and who have not used illicit drugs support the implementation of pill testing services. People would change their consumption patterns according to the results given by pill testing services and are also in favour of pill testing services combined with harm reduction advice. Increased frequency of MDMA usage is associated with increased likelihood of taking a pill despite pill testing services detecting a harmful substance. The findings may be used to stimulate public debate, and assist drug and alcohol policy makers in the implementation of harm minimisation strategies such as combining pill testing services with harm reduction advice.

## Supplementary information

**Additional file 1.** Survey questions. Survey questions. Survey questions.

## Data Availability

The datasets generated and/or analysed during the current study are not publicly available due to the existing ethics approval.

## Abbreviations

2C-B/CA: 2,5-Dimethoxy-4-bromophenethylamine
AUDIT: Alcohol use disorders identification test
CI: Confidence interval
DF: Degrees of freedom
MDA: Methylenedioxyamphetamine
EDRS: Ecstasy and related drugs reporting system
DOB: Dimethoxybromoamphetamine
DOI: 2,5- Dimethoxy-4-iodoamphetamine
DXM: Dextromethorphan
GHB: Gamma hydroxybutyrate
OR: Odds ratio
MDMA: 3,4-Methylenedioxymethamphetamine
mCPP: meta-Chlorophenylpiperazine
NSW: New South Wales
PASH: Positive adolescent sexual health
PMA: Paramethoxyamphetamine
RSA: Responsible Service of Alcohol
