# Supplementary material for: Patterns of ecstasy use amongst live music event attendees and their opinions on pill testing: a cross sectional study
Source: Subst Abuse Treat Prev Policy. 2020 Aug 5;15:55. doi: 10.1186/s13011-020-00295-1 (PMC7405356; doi:10.1186/s13011-020-00295-1)
Supplement: Supplementary file 1 — Additional file 1. Survey questions. Survey questions. Survey questions. [file 13011_2020_295_MOESM1_ESM.pdf]

Pill Testing at Music Festivals Survey

This survey is being conducted to gain insight into the opinion of music festival attendees regarding pill-testing programmes. This survey is anonymous and confidential. The survey should take no longer than 5 minutes to complete and you may withdraw at any time until the survey is submitted. Your time would be most appreciated.

1. ***Do you identify as:*** ☐ Male ☐ Female ☐ Other (please specify)_________________
2. ***Your age:*** ______
3. ***Your postcode:*** _____________
4. ***Are you:*** ☐ Married/de facto ☐ Single ☐ In a relationship ☐ Separated/Divorced/Widowed
5. ***Are you:*** ☐ Heterosexual ☐ Homosexual ☐ Bisexual ☐ Other
6. ***Are you:*** ☐ A fulltime student ☐ A part-time student ☐ Employed ☐ Unemployed ☐ Other
7. ***On average, how many hours A WEEK do you undertake <u>paid</u> employment?*** ____________ hours
8. ***How often do you have a drink containing alcohol?***

Never	Monthly or less	2-3 times a month	2-3 times a week	4 or more times a week
☐	☐	☐	☐	☐

9. ***On a typical day that you drink, how many standard drinks containing alcohol do you consume?***

   1 or 2 ☐ 3 or 4 ☐ 5 or 6 ☐ 7 to 9 ☐ 10 or more ☐

10. ***How often do you have six or more standard drinks on one occasion?***

Never	Less than monthly	Monthly	Weekly	Daily or almost daily
☐	☐	☐	☐	☐

Pill testing services could test drugs by taking a small sample. A reagent testing kit is used to test this sample, gathering information on its contents. The user can then be informed of the presence of potentially harmful ingredients/substances.

11. ***How much do you agree with the following statements:***

		**Not at all**	**A Little**	**Somewhat**	**A lot**
a.	Pill testing should be provided for FREE at music festivals	☐1	☐2	☐3	☐4
b.	Pill testing should be provided at a cost at music festivals	☐1	☐2	☐3	☐4
c.	Pill testing services could help drug users seek help to reduce harm	☐1	☐2	☐3	☐4
d.	Pill testing services should be combined with harm reduction advice	☐1	☐2	☐3	☐4
e.	Drug sellers may use the service as a quality control mechanism	☐1	☐2	☐3	☐4
f.	I would be more likely to take illicit drugs at a music festival if pill-testing services were present	☐1	☐2	☐3	☐4

12. ***Do you have any concerns about pill testing at music festivals? Please explain:***

____

____

____

13. **Have you ever taken illicit drugs?**

    ☐ Yes ☐ No

If you answered <u>No</u> this is the end of the survey. If you answered <u>Yes</u> please complete the sections on the next page

PLEASE TURN OVER

© Pit, S.W. et al. 2017.

14. ***In the last 12 MONTHS, have you used any of the drugs listed below (on at least one occasion)? Tick as many as apply***

☐ Alcohol	☐ Methamphetamine	☐ GBH	☐ Synthetic cannabis
☐ Tobacco	☐ Ecstasy/MDMA	☐ Hallucinogens	☐ Inhalants
☐ Amphetamine (speed)	☐ Cocaine	☐ Recreational Pharmaceutical drugs	☐ Other (Please specify) ___________________
☐ Cannabis	☐ Ketamine	☐ Steroids	☐ None

15. ***Over the last 12 months, how many days have you:***

a) Taken time off from work/study because of **drug use** (e.g. coming down)? _____________ days

b) Gone to work/study despite feeling that you should have taken sick leave because of using illicit drugs? __________ days

16. ***Have you ever consumed MDMA/ecstasy at a music festival?***

☐ Yes ☐ No

17. ***Have you ever mixed MDMA/ecstasy with other substances at a music festival?***

☐ Yes ☐ No

a) If Yes, what other substances?

____

18. ***How much MDMA/ecstasy do you take at one time at a music festival?***

____

19. ***How often do you use MDMA/ecstasy?***

☐ Daily ☐ Weekly ☐ Fortnightly ☐ Monthly

☐ 6 Months ☐ Yearly ☐ Once ☐ Never

20. ***Have you ever had to seek medical attention due to taking MDMA/Ecstasy?***

☐ Yes ☐ No

21. ***Please answer the following questions:***

		Not at all	A Little	Somewhat	A lot
a.	In terms of your health, how concerned are you about the content and/or purity of the illicit drugs you take?	☐1	☐2	☐3	☐4
b.	If a harmful substance was detected in your drugs using the pill testing service, how likely would you be to still consume them?	☐1	☐2	☐3	☐4
c.	Currently, how likely are you to attempt to find out about the content and/or purity of the illicit drugs you intend to take?	☐1	☐2	☐3	☐4
d.	How likely is it that you would use a **FREE** pill testing service?	☐1	☐2	☐3	☐4

22. ***Would you still take a drug if the results of a drug testing kit/ pill testing service indicated the presence of the following unintended drugs?***

	Yes	No	Don't know
MDMA-type substances (MDA, MDE)	☐1	☐2	☐3
Amphetamine	☐1	☐2	☐3
Methamphetamine	☐1	☐2	☐3
Ketamine	☐1	☐2	☐3
DXM	☐1	☐2	☐3
2C-B/C/I	☐1	☐2	☐3
DOB	☐1	☐2	☐3
DOI	☐1	☐2	☐3
Methylone	☐1	☐2	☐3
Butylone	☐1	☐2	☐3
Naphyrone	☐1	☐2	☐3
Opiates	☐1	☐2	☐3
PMA/PMMA	☐1	☐2	☐3
Other (please specify)	☐1	☐2	☐3
No reaction (benign or unknown substances)	☐1	☐2	☐3

23. ***If the pill testing service was not free, how much would you pay to test your pills?***

____

24. ***Do you have any other comments?***

____

Thank you for your time and cooperation! **PLEASE PUT SURVEY IN THE BOX**

If completion of this survey has caused any distress, please consider the following options: Contact your own GP or Helpline on: 13 11 14 or visit http://www.beyondblue.org.au to identify other sources of help.
